# Supplementary material for: Early Paleoindian use of canids, felids, and hares for bone needle production at the La Prele site, Wyoming, USA
Source: PLoS One. 2024 Nov 27;19(11):e0313610. doi: 10.1371/journal.pone.0313610 (PMC11602046; doi:10.1371/journal.pone.0313610)
Supplement: S1 File — (PDF) [file pone.0313610.s001.pdf]

# Supporting Information for

# Early Paleoindian use of canids, felids, and hares for bone needle production at the La Prele site, Wyoming, USA

Spencer R. Pelton[1]*, McKenna Litynski[2], Sarah A. Allaun[3], Michael Buckley[4], Jack Govaert[5], Todd Schoborg[5], Matthew O'Brien[6], Matthew G. Hill[7], Paul Sanders[1], Madeline E. Mackie[8], Robert L. Kelly[2], Todd A. Surovell[2]

*1000 East University Ave, Dept 3431, Laramie, WY 82071; 307-399-2827; spencer.pelton@wyo.gov

[1]Office of the Wyoming State Archaeologist, University of Wyoming, Laramie, Wyoming 82071

[2]Department of Anthropology, University of Wyoming, Laramie, Wyoming 82071

[3]History Colorado Office of Archaeology and Historic Preservation, Denver, Colorado 80203

[4]School of Natural Sciences, Manchester Institute of Biotechnology, University of Manchester, Manchester, UK M13 9PL

[5]Department of Molecular Biology, University of Wyoming, Laramie, Wyoming 82071

[6]Department of Anthropology, California State University Chico, Chico, California 95929

[7]World Languages and Cultures, Iowa State University, Ames, Iowa 50011

[8]Department of Anthropology, Michigan State University, East Lansing, Michigan 48824

Spencer R. Pelton

Email: spencer.pelton@wyo.gov

**This PDF file includes:**

Figures S1 to S3

Table S1

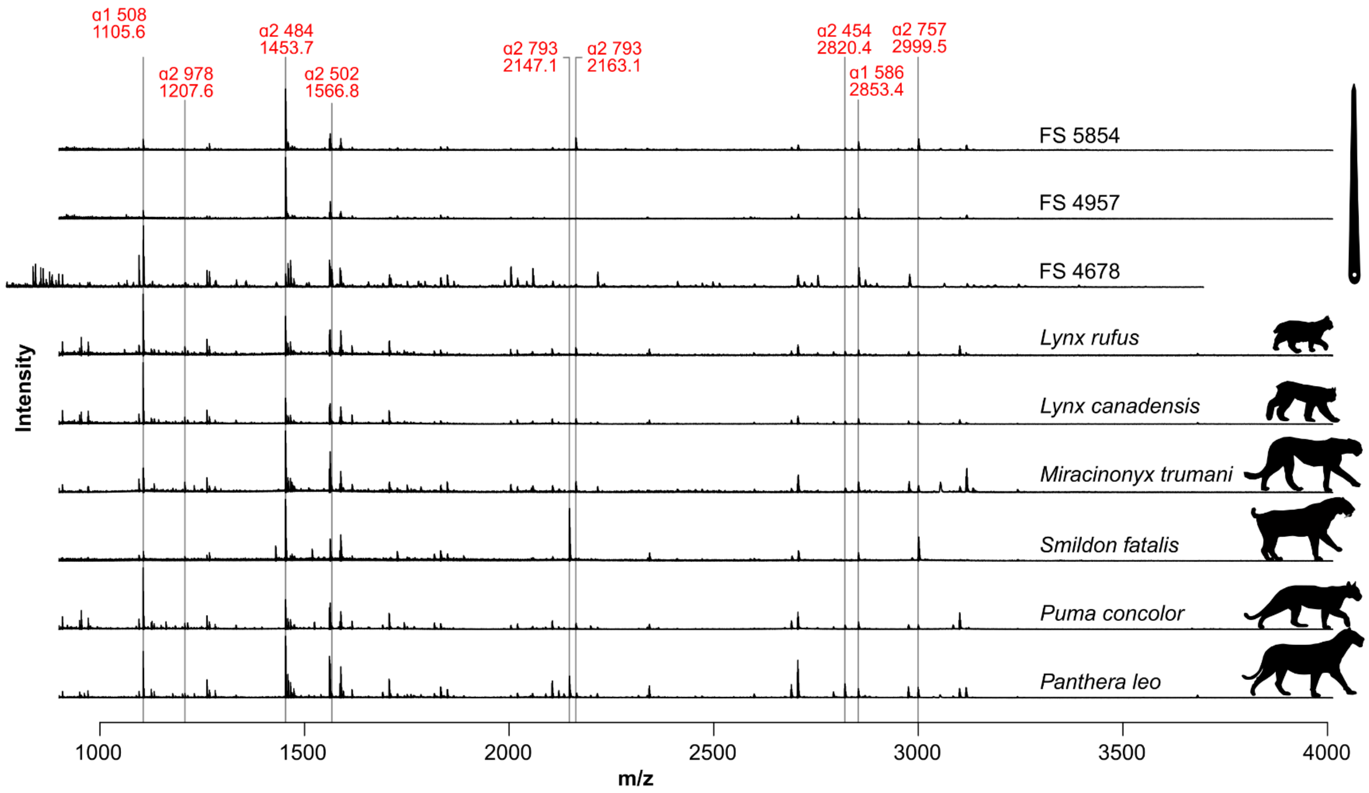


Fig. S1. MALDI-TOF spectra of needles identified as having been manufactured with felid bone in comparison to extant and extinct candidate felid taxa.

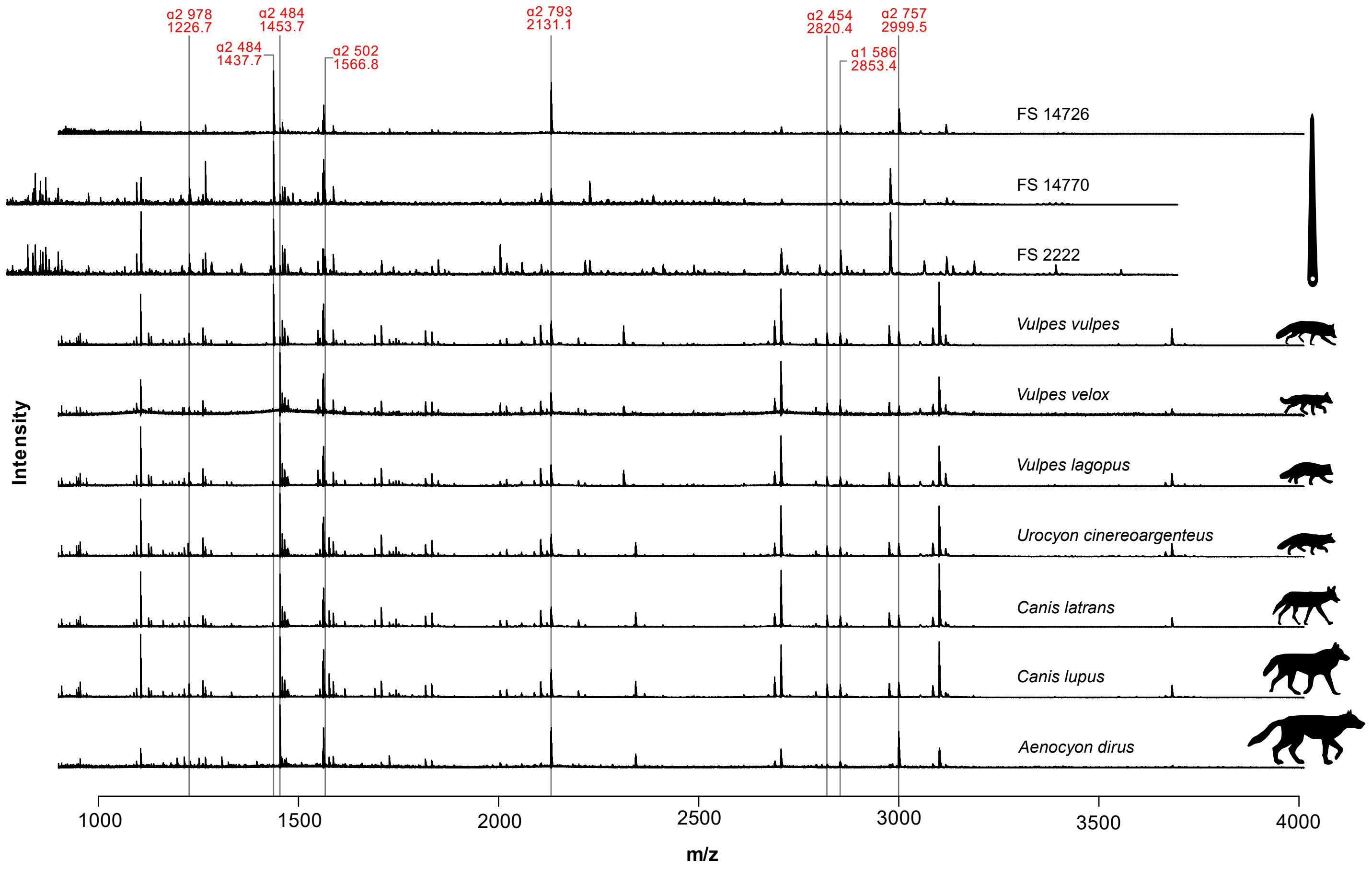


Fig S2. MALDI-TOF spectra of needles identified as having been manufactured with canid bone in comparison to extant and extinct candidate canid taxa.

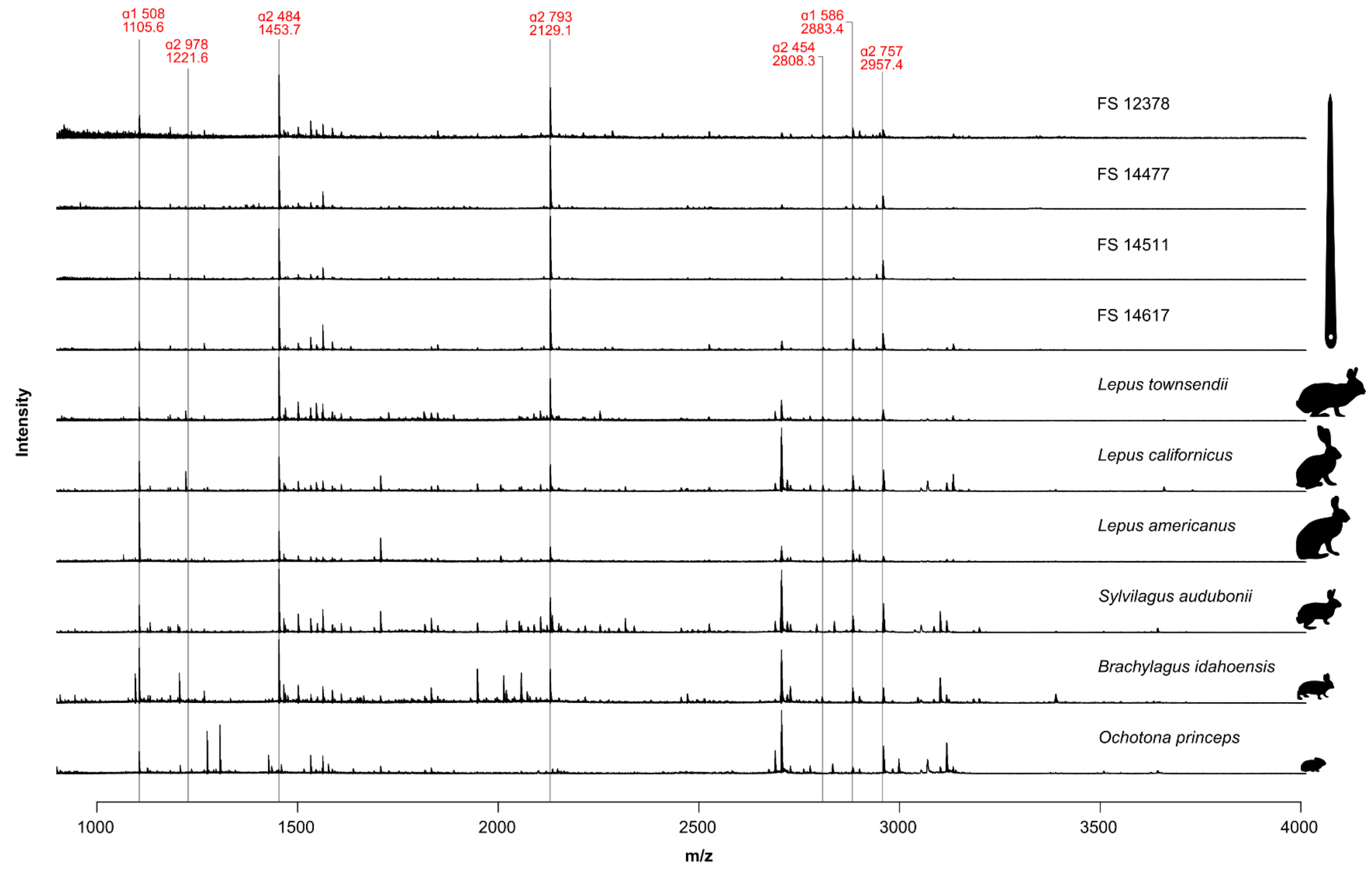


Fig S3. MALDI-TOF spectra of needles identified as having been manufactured with leporid bone in comparison to extant and extinct candidate lagomorph taxa.

| | α2 978 | | | α2 484 | | α2 502 | α2 793 | | | | α2 454 | | | α1 586 | | α2 757 | | | |
|---|---|---|---|---|---|---|---|---|---|---|---|---|---|---|---|---|---|---|---|
| **Artifact or Species** | **1207.6/ 1223.6** | **1210.7/ 1226.7** | **1221.6/ 1235.6** | **1437.7** | **1453.7** | **1566.8** | **2129.1** | **2131.1** | **2147.1** | **2163.1** | **2808.3** | **2820.4** | **2836.3** | **2853.4/ 2869.4** | **2883.4/ 2899.4** | **2957.4/ 2973.5** | **2983.5/ 2999.5** | **Source** | **Ident-ification** |
| FS 4957 | | | | | X | X | | | | X | | X | X | X | | | X | | Family: *Felidae* |
| FS 5854 | | | | | X | X | | | | X | | X | X | X | | | X | | Family: *Felidae* |
| FS 12378 | | X | X | | X | X | X | | | | X | | | | X | X | | | Genus: Lepus |
| FS 14477 | | | X | X | X | X | X | | | | X | | | | X | X | | | Genus: Lepus |
| FS 14511 | | | X | X | X | X | X | | | | X | | | | X | X | | | Genus: Lepus |
| FS 14617 | | | X | X | X | X | X | | | | X | | | | X | X | | | Genus: Lepus |
| FS 14726 | | ? | | X | X | X | | X | | | | X | | X | | | X | | *Vulpes vulpes* |
| FS 14770 | ? | X | | X | X | X | | X | | | | ? | | X | | | X | | *Vulpes vulpes* |
| FS 2222 | X | X | | X | X | X | | X | | | | X | | X | X | | X | | *Vulpes vulpes* |
| FS 4678 | X | | | | X | X | | | | X | | | | X | X | | X | | Family: *Felidae* |
| *Lynx Rufus* | X | | | | X | X | | | | X | | X | | X | | | X | 2 | |
| *Lynx Canadensis* | X | | | | X | X | | | | X | | X | | X | | | X | 2 | |
| *Miracinonyx trumani* | X | | | | X | X | | | | X | | X | X | X | | | X | this study | |
| *Puma concolor* | X | | | | X | X | | | ? | X | | X | | X | | | X | this study | |
| *Smilodon fatalis* | X | | | | X | X | | | X | ? | | | | X | | | X | this study | |
| *Panthera leo* | X | | | | X | X | | | X | | | X | X | X | | | X | 1 | |
| *Vulpes lagopus* | | X | | | X | X | | X | | | | X | X | X | | | X | 2 | |
| *Vulpes vulpes* | | X | | X | X | X | | X | | | | X | X | X | | | X | 2 | |
| *Vulpes velox* | | X | | | X | X | | X | | | | X | | X | | | X | this study | |
| *Urocyon cinereoargenteus* | | | | | X | X | | X | | | | X | X | X | | | X | this study | |
| *Canis latrans* | | X | | | X | X | | X | | | | X | X | X | | | X | 2 | |
| *Canis lupus* | | X | | | X | X | | X | | | | X | X | X | | | X | 2 | |
| *Aenocyon dirus* | | | | | X | X | | X | | | | X | ? | X | | | X | this study | |
| *Lepus europaeus* | | | X | | X | | X | | | | X | | | | X | X | | 1 | |
| *Lepus californicus* | | | X | X | X | X | X | | | | X | | | | X | X | | this study | |
| *Lepus americanus* | | | X | X | X | X | X | | | | X | | | | X | X | | this study | |
| *Lepus townsendii* | | | X | X | X | X | X | | | | X | | | | X | X | | this study | |
| *Sylvilagus audubonii* | | | X | X | X | X | X | | | | | | X | | X | X | | this study | |
| *Brachylagus idahoensis* | | | X | X | X | X | X | | | | | | | | X | X | | this study | |
| *Ochotona princeps* | | | X | | X | | | | X | X | | | | | X | | | this study | |

*Notes: 1. M. Buckley, M. Collins, J. Thomas-Oates, J. C. Wilson, Species identification by analysis of bone collagen using matrix-assisted laser desorption/ionisation time-of-flight mass spectrometry. Rapid Communications in Mass Spectrometry: An International Journal Devoted to the Rapid Dissemination of Up-to-the-Minute Research in Mass Spectrometry 23, 3843–3854 (2009). 2. F. Welker, M. Hajdinjak, S. Talamo, K. Jaouen, M. Dannemann, F. David, M. Julien, M. Meyer, J. Kelso, I. Barnes, S. Brace, P. Kamminga, R. Fischer, B. M. Kessler, J. R. Stewart, S. Pääbo, M. J. Collins, J.-J. Hublin, Palaeoproteomic evidence identifies archaic hominins associated with the Châtelperronian at the Grotte du Renne. Proc. Natl. Acad. Sci. U.S.A. 113, 11162–11167 (2016).*

Table S1. Presence and absence of marker peptides for needles, needle preforms, and comparative canid, leporid, and felid taxa
