# Supplementary material for: Early Paleoindian use of canids, felids, and hares for bone needle production at the La Prele site, Wyoming, USA
Source: PLoS One. 2024 Nov 27;19(11):e0313610. doi: 10.1371/journal.pone.0313610 (PMC11602046; doi:10.1371/journal.pone.0313610)
Supplement: Attachment — Submitted filename: Response to Reviewers.docx [file pone.0313610.s002.docx]

1. Please ensure that your manuscript meets PLOS ONE's style requirements, including those for file naming. The PLOS ONE style templates can be found at

<https://journals.plos.org/plosone/s/file?id=wjVg/PLOSOne_formatting_sample_main_body.pdf> and

<https://journals.plos.org/plosone/s/file?id=ba62/PLOSOne_formatting_sample_title_authors_affiliations.pdf>

We modified several aspects of the manuscript according to these style requirements, including reformatting Figure and Table captions and references, changing the heading font size, modifying aspects of the title page, adding supplemental captions to end of manuscript, and reformatting some citations, among other minor changes.

2. In your manuscript, please provide additional information regarding the specimens used in your study. Ensure that you have reported human remain specimen numbers and complete repository information, including museum name and geographic location.

We added this sentence to the beginning of the methods section: *The artifacts used in this study were recovered from archaeological excavations by the University of Wyoming Department of Anthropology between 2015 and 2022 from the La Prele site, located on private land in Converse County, Wyoming. The artifacts are housed at the University of Wyoming in the lab of Todd Surovell.*

If permits were required, please ensure that you have provided details for all permits that were obtained, including the full name of the issuing authority, and add the following statement:

'All necessary permits were obtained for the described study, which complied with all relevant regulations.'

If no permits were required, please include the following statement:

'No permits were required for the described study, which complied with all relevant regulations.'

For more information on PLOS ONE's requirements for paleontology and archeology research, see <https://journals.plos.org/plosone/s/submission-guidelines#loc-paleontology-and-archaeology-research>.

No permits were required. We added the second statement to the materials and methods section.

3. Thank you for stating the following financial disclosure:

“Funding for this project includes the National Science Foundation, the Wyoming Cultural Trust Fund, the Quest Archaeological Research Program at Southern Methodist University, and the George C. Frison Institute for Archaeology and Anthropology.”

Please state what role the funders took in the study.  If the funders had no role, please state: "The funders had no role in study design, data collection and analysis, decision to publish, or preparation of the manuscript."

If this statement is not correct you must amend it as needed.

Please include this amended Role of Funder statement in your cover letter; we will change the online submission form on your behalf.

4. Thank you for stating the following in the Acknowledgments Section of your manuscript:

“The Strock family has been gracious in allowing investigations of La Prele on their land. Funding for this project includes the National Science Foundation, the Wyoming Cultural Trust Fund, the Quest Archaeological Research Program at Southern Methodist University, and the George C. Frison Institute for Archaeology and Anthropology.”

We note that you have provided funding information that is currently declared in your Funding Statement. However, funding information should not appear in the Acknowledgments section or other areas of your manuscript. We will only publish funding information present in the Funding Statement section of the online submission form.

We removed this from the acknowledgements.

Please remove any funding-related text from the manuscript and let us know how you would like to update your Funding Statement. Currently, your Funding Statement reads as follows:

“Funding for this project includes the National Science Foundation, the Wyoming Cultural Trust Fund, the Quest Archaeological Research Program at Southern Methodist University, and the George C. Frison Institute for Archaeology and Anthropology.”

Please include your amended statements within your cover letter; we will change the online submission form on your behalf.

In accordance with your request, please amend the funding statement to read:

*Funding for this project includes the National Science Foundation, the Wyoming Cultural Trust Fund, the Quest Archaeological Research Program at Southern Methodist University, and the George C. Frison Institute for Archaeology and Anthropology. The funders had no role in study design, data collection and analysis, decision to publish, or preparation of the manuscript.*

5. Please review your reference list to ensure that it is complete and correct. If you have cited papers that have been retracted, please include the rationale for doing so in the manuscript text, or remove these references and replace them with relevant current references. Any changes to the reference list should be mentioned in the rebuttal letter that accompanies your revised manuscript. If you need to cite a retracted article, indicate the article’s retracted status in the References list and also include a citation and full reference for the retraction notice.

We made a substantial number of edits to the reference section to account for errors incurred by the citation management system. This included removing a duplicate reference.

Additional Editor Comments:

This is an excellent study that will be of interest to many researchers. The two reviews of this paper are both very positive and raise only minor questions and suggest small edits. The two reviews conflict on the necessity of the description in the Material and Methods section, with Reviewer 1 suggesting it could be moved to Supplemental materials and Reviewer 2 requesting the addition of some detail in this section. My inclination is to keep the Materials and Methods section as is and perhaps address Reviewer 2's concern.

5. Review Comments to the Author

Reviewer #1: What a nifty paper!! Really solid analyses and logic that not only provide insight to which animal taxa were exploited for what purposes but reminds us quite explicitly (and correctly) that animals were more than food sources for foragers. I like this paper a lot and find it quite worthy of publication in PLOS One. However, before publication there are some things that need to be fixed; these are mostly typographical errors and points of clarification, nothing terribly onerous. Taking them in order in the manuscript:

--p. 3, last paragraph: “like canids, felids, etc”. which implies not canids, felids, etc. but something “like” them, so change “like” to “such as”

We made this change

--p. 3, last paragraph, sentence spanning lines 4-6: may want to add that carnivores are relatively rare on the landscape whereas leporids are typically relatively abundant

We made this change

--p. 3, last paragraph, third line from bottom: “the first evidence” when I initially read this, my thought was hardly that this was “the first” as others had made similar arguments, but it is of course the first discussion of La Prele needles (so far as I know). But think about deleting “the first” or rewording so the wrong impression is not suggested.

We deleted “the first”

--p. 4, first paragraph under “Materials and Methods”, last sentence: why measured in five locations? Even on specimens a few mm long? And how are these data reflected in Fig. 4? Of what significance is taking measurements in five locations on a specimen, even if it is a complete needle? Without answering these questions, this is information that adds nothing but words to the manuscript.

We added the following sentence: *We measured each specimen in five locations to characterize the range of variation present within each specimen, regardless of the specimen’s total size.*

--pp. 4-6: “Materials and Method”— I find much of these paragraphs to be unnecessary. ZooMS is a well established, well known and generally accepted viable method, and Micro-CT scanning seems similarly known among at least some people. These paragraphs distract one’s attention from the focus of the article—which taxa contributed their bones to Paleoindian needles? I suggest considering putting much of this in supplemental material, or shortening it considerably.

We deferred to reviewer 2 and the Editor on this. We included one additional sentence per reviewer 2 but left the rest of this section intact.

--p. 5, second paragraph: these taxonomic names should include the common names here (on first mention) as well. Will most readers know what a Miracinonyx trumani is? Or what a Uroycyon cinereoargenteus is? Once common names have been listed, any later mention of specific taxa can just be to taxonomic names or common names (one or the other). Listing both taxonomic and common names later (such as found on p. 6 and p. 8 and p. 11) is too late to be helpful (and duplication is unnecessary). See also my comment on p. 7 below.

We added common names to this section in parentheses.

--p. 6, next to last line: “std” I suspect I know what this is--it likely is not “sexually transmitted disease”. But from my perspective it is not standard statistical abbreviation; SD is what I am used to seeing (assuming what is meant is “standard deviation”). Same comment for second line on p. 7.

We changed all mention of std to SD.

--p. 7, table 1: why are both species and common names listed? I suggest using one or the other rather than the mixture. See my comment on p. 5 above.

The ZooMS ID field in this Table contains only the genus. We have added that to the field header and italicized the column to better denote this.

--p. 8, last paragraph, third line: “sp.” should not be italicized here or anyplace else.

We removed italics from every instance of this in the paper.

--p. 8, last line: “poorly understood”. This may be true in Wyoming but not everywhere where pygmy rabbit is found (e.g., Lyman, RL. 2004. Biogeographic and Paleoenvironmental Implications of Late Quaternary Pygmy Rabbits (Brachylagus idahoensis) in Eastern Washington. Western North American Naturalist 64:1–6). Does perhaps the most recent rendition of FAUNMAP have some info on this?

We added the words “in Wyoming” to this sentence. We did a query for the species in Neotoma, and it didn’t produce any results for Wyoming. Our impression is that pygmy rabbits are primarily a Great Basin species and we simply haven’t recovered any from an archaeological or paleontolological context in SW Wyoming.

--p. 9, line 4: “four bone needles”. I suspect, based on subsequent discussion, this is much like a “minimum number” estimate based on similarity in dimensions, provenience, and taxon represented by sets of fragments. It would help to state such bases for the estimate here rather than leaving it until later.

We added the phrase “minimum number of” to this sentence. We kind of already explain how we derived these numbers in a general sense at the end of this sentence, which ends with “based on a combination of their morphological characteristics, spatial distributions, and ZooMS results”

--p. 9, just above figure 3 caption: “Lynx/Puma/Miracinonyx” all should be italicized

We italicized this.

--p. 10, second paragraph, line 3: “5 to 7 cm long”—how many specimens are complete or nearly so? As I recall from Lyman (2015), most specimens are shorter but are incomplete. And think about this (as Lyman 2015 suggests), if the needle is two long, it likely will snap as one attempts to wiggle it back and forth through a too-small hole already in the hide. Why do you suppose so many of the needles we find are broken? I doubt this is all post-depositional fragmentation.

We rephrased this sentence to read: *Although none of the bone needles from La Prele are complete as a result of use-related (18) and post-depositional fracturing, rare examples of mostly complete bone needles of comparable age from the Agate Basin (41) and Lindenmeier (42) sites are 5 to 7 cm long and 1.5 to 2.5 mm thick.*

--p. 10, second paragraph, last four lines: lengthwise split leporid metapodials. Not an unreasonable suggestion, but have you tried to split one of them lengthwise? I suspect it would not be easy to do it consistently successfully. How, for instance, is the metapodial firmly secured such that graving its length (seems the most logical technique of splitting) doesn’t skitter all over?

We changed this sentence to: *One would need only to score metapodials lengthwise with a flake or graving tool, split them, and then pare down their width through carving or abrading to achieve appropriate dimensions for bone needles.*

--p. 11, fourth line from bottom: this took me a few minutes to figure out what the “two artifacts” are: the bone needle artifact was easy to note, but the “fur-bearing mammal bone” as an artifact was not, likely because 99.3% of all zooarchaeological mammal bones I have ever studied were NOT considered artifacts by the archaeologists who dug them up and sent them to me. And this is my impression of the discipline at large—mammal bones ain’t artifacts (if anything other than animal bones, they are ecofacts). Consider rewording this sentence.

We reworded this sentence to read: *At La Prele, the production of bone needles from fur-bearing mammal bone most likely occurred because both bone needles and fur-bearer pelts were used to produce tailored garments.*

--p. 12, lines 4-5: you might consider noting that the trapping notion has a test implication. Trapped carnivorous fur bearers tend to be dominated by males rather than females given behavioral differences (for references and an archaeological example, see: Lyman, R. L. 2007. Prehistoric Mink (Mustela vison) Trapping on the Northwest Coast (USA). Journal of Field Archaeology 32:91–95). Not that you need to do this, but it might be good to mention this as an avenue of future research.

We added these two sentences and additional citation to the end of this paragraph: *A further expectation of trapping is an overrepresentation of males among fur-bearing carnivores in archaeological sites, which are more likely to be trapped given behavioral differences (52). Sex determination of fur bearing carnivores in Paleoindian assemblages would thus be an interesting avenue for future research.*

--p. 12, line 3 of conclusions: “entirely conjectural” seems an overstatement as it implicitly derogates and belittles experimental work. Experimental work of course does not show how things were done in the past, only how they may have been done efficiently and effectively. But to categorize that work as conjectural will no doubt offend lithic replicators and any other experimental archaeologist. To then turn around and say “we obtained these results” is not only confusing (which results? On manufacturing protocol?) but misleading as it implies you did something regarding “bone needle production techniques” that previous workers did not do. In fact, you only determined which taxa were exploited; you did not determine how the needles were manufactured. And, you only conjectured that leporid metapodials were split lengthwise, and you did not even do experimental work to show that such was possible or whether grinding/abrasion or whittling or whatever was used to finish the needle. And you mention no evidence on the archaeological specimens as to whether they were ground/abraded or whittled or what.

We edited this sentence and added an additional citation to another bone needle production experiment: *Bone needle production techniques have until this point been largely theoretical, based on incised bone objects argued to represent bone needle preforms (53,54) and experimental needle production (55,56).*

--p. 13-17: there are typographical errors (e.g., refs 1 &19), formatting inconsistencies (e.g., sometimes book titles are capitalized, sometimes not), and missing info (e.g., refs. 28 & 30 & 34) scattered among the references. I have not listed them all here.

The list of things I believe require attention may seem long, but as indicated above, they are all relatively minor things. They do, nevertheless, need to be addressed. Once they are, this will be a significant contribution and I look forward to seeing a revised version published. I wish to be named as referee: R. Lee Lyman

Reviewer #2: This is an excellent manuscript and addresses an area worthy of investigation. What's more, the data and arguments are presented clearly and concisely.

The paper would be improved by addressing/clarifying two minor issues. First, there are frequent references to prey choice models and the place of furbearers in those models. In the text it isn't clear if the traditional return rates on furbearers (and thus their rank in traditional diet breadth models) are based on hunting or trapping. I don't think this changes the outcome of the study in any way but it would clarify whether or not the trapping behavior proposed in the article would affect the rank of these resources in existing prey choice models.

This is an interesting issue, but one that I am afraid anthropologists don’t have a great answer to. There are few to no documented instances of encounter hunting of these animals, so we don’t know how it might compare to trapping, for which we have some examples. We clarified this by adding the following sentence to the discussion: *In a recent review of all well-established return rates in the ethnographic literature, Morin and colleagues (24) document relatively few fur bearers, and those present were invariably trapped or hunted in game drives rather than pursued in a solo hunting scenario. Given that ethnographic cases of solo, encounter-based hunting for fur bearers appear to be non-existent, we must assume that such animals were rarely if ever obtained in ways other than trapping or driving (in the case of lagomorphs).*

Second, the methods indicate that taxonomic identification relied on published marker peptides plus additional taxa analyzed for this study. What isn't clear is whether the published marker peptides included spectra for mustelids, procyonids, and other small mammals that could potentially be represented, or whether these other taxa were eliminated by other means.

We added the following sentence to the end of the methods section: *Mustelidae and Procyonidae can be eliminated as candidate taxa based on the absence of the 1219.6/1235.6 α2 978  peptide pair in all needle spectra.*

Perhaps these issues go without saying to someone more familiar with Zooms, but the average reader (like this reviewer) might benefit from clarification. Both of these issues could be easily clarified in an added sentence or two.

Well done, very interesting, and a worthwhile contribution.
